# Impact of surface coating and systemic anticoagulants on hemostasis and inflammation in a human whole blood model

**DOI:** 10.1371/journal.pone.0280069

**Published:** 2023-01-12

**Authors:** Doreen Tabea Spiegelburg, Marco Mannes, Anke Schultze, Frieder Scheibenberger, Frederik Müller, Amadeo Klitzing, David Alexander Christian Messerer, Kristina Nilsson Ekdahl, Bo Nilsson, Markus Huber-Lang, Christian Karl Braun

**Affiliations:** 1 Institute for Clinical and Experimental Trauma Immunology, University Hospital of Ulm, Ulm, Germany; 2 Department of Transfusion Medicine and Hemostaseology, University Hospital of Erlangen, Friedrich-Alexander University Erlangen-Nürnberg (FAU), Erlangen, Germany; 3 Centre of Biomaterials Chemistry, Linnaeus University, Kalmar, Sweden; 4 Rudbeck Laboratory, Department of Immunology, Genetics and Pathology, Uppsala, Sweden; Universidade Federal do Rio de Janeiro, Instituto de Bioquimica Medica, BRAZIL

## Abstract

**Background:**

Surface compatibility with blood is critical both for scientific investigations on hemostasis and clinical applications. Regarding in vitro and ex vivo investigations, minimal alteration in physiological hemostasis is of particular importance to draw reliable conclusions on the human coagulation system. At the same time, artificial coagulation activation must be avoided, which is relevant for the patient, for example to prevent stent graft occlusion. The aim was to evaluate the advantages and disadvantages of antithrombotic and antifouling surface coatings in the context of their suitability for ex vivo incubation and the study of coagulation properties.

**Methods:**

We investigated the impact of different protocols for surface coating of synthetic material and different anticoagulants on hemostasis and platelet activation in ex vivo human whole blood.

Blood samples from healthy donors were incubated in coated microtubes on a rotating wheel at 37°C. Two protocols for surface coating were analyzed for hemostatic parameters and metabolic status, a heparin-based coating (CHC, Corline Heparin Conjugate) without further anticoagulation and a passivating coating (MPC, 2-methacryloyloxethyl phosphorylcholine) with added anticoagulants (enoxaparin, ENOX; or fondaparinux, FPX). Employing the MPC-based coating, the anticoagulants enoxaparin and fondaparinux were compared regarding their differential effects on plasmatic coagulation by thrombelastometry and on platelet activation by flowcytometry and platelet function assays.

**Results:**

Using the CHC coating, significant coagulation cascade activation was observed, whereas parameters remained mostly unchanged with MPC-based protocols. Extended incubation caused significantly elevated levels of the soluble membrane attack complex. Neither ENOX nor FPX caused a relevant impairment of platelet function or activation capacity and thrombelastometric parameters remained unchanged with both protocols. For translational purposes, we additionally modeled endotoxemia with the MPC-based protocols by incubating with lipopolysaccharide plus/minus thrombin. While coagulation parameters remained unchanged, elevated Interleukin 8 and Matrix Metalloproteinase 9 demonstrated preserved immune cell responsiveness.

**Conclusions:**

The MPC-based protocols demonstrated better hemocompatibility compared to CHC, and ENOX and FPX proved useful for additional anticoagulation. Furthermore, this simple-to-use whole blood model may be useful for experimental analyses of the early coagulatory and immunological response without decalcification.

## Introduction

In clinical practice, biocompatibility plays an important role, for example with stent grafts and in hemodialysis and extracorporeal membrane oxygenation (ECMO) [1–3]. Advancing these associated materials regarding their immune- and coagulation-modulating properties upon exposure with blood remains part of current research. Therefore, improvements should aim to prevent protein adhesion, cell deposition and protease activation on the artificial surfaces to mimic physiological conditions and to further improve the patient’s outcome [3].

Concerning hemostasis, bleeding and thrombosis are among the main complications observed in patients requiring ECMO [4,5] due to multifactorial events like the depletion of coagulation factors, excessive anticoagulation and/or thrombocytopenia [6]. Of note, Krueger et al. performed ECMO on patients without heparinization, and solely provided the low-molecular weight heparin (LMWH) enoxaparin (ENOX) as a thrombosis prophylaxis [6]. Following their findings, other groups demonstrated reduced bleeding events without pharmacological anticoagulation and with heparin-based surface-coating only [7], and also reduced thrombotic events by changing from unfractionated heparin (UFH) to LMWH [8]. Evaluation of different regimes of anticoagulation and better surface compatibility of biomaterials appears essential to improve the safety profile of extracorporeal blood circulation. However, approaches using in-vivo animal models are often hardly transferable to human coagulation physiology [9]. Therefore, experimental studies are required which readily and reliably analyze human whole blood with minimal changes in blood physiology, cell metabolism, cell-cell interactions and innate immune performance.

To improve hemocompatibility of artificial surfaces, one approach is to apply an antifouling surface coating like the 2-methacryloyloxethyl phosphorylcholine (MPC)-based polymer. MPC coating results in reduced blood cells adhesion and less surface-driven activation of the complement and coagulation systems [10–12]. Some of the reported effects are caused by a reduced adsorption of fibrinogen to the material [13], which inhibits one of the main mechanisms leading to platelet adhesion [14]. Another concept is to coat foreign material with unfractionated heparin to prevent surface activation. Such heparin coating protocols may lead to less platelet and leukocyte activation [15]. A heparin-based commercially available surface-coating (Corline Heparin Conjugate; CHC) has already been tested on patients who underwent coronary artery bypass surgery [16]. In the case of extracorporeal circulation, systemic anticoagulation is needed to prevent thrombosis, however, with the increased risk for bleeding events [17,18]. It was described previously that LMWH and the pentasaccharide fondaparinux (FPX) lead to reduced effects on the prothrombin time compared to UFH [19]. Nevertheless, FPX is more commonly used as an alternative for patients with contraindications for UFH [20].

The aim of this study was to compare three different protocols for an ex vivo modeling of hemostasis regarding their ability to achieve “steady state” conditions with the prevention of artificial activation of the coagulation system and synchronous maintenance of the hemostatic function of human whole blood. We used an established protocol employing an antithrombogenic coating with UFH (without adding further anticoagulants to the blood samples) and an approach using the previously described passivating MPC coating with either ENOX or the heparinoid FPX.

## Material and methods

### Blood sample preparation

Blood was donated by healthy adult volunteers of both sexes without current medication after they provided written informed consent. All experiments were approved by the independent ethics committee of Ulm University (Application-No. 462/18 and Application-No. 103/20).

For the CHC-based model, venous blood was drawn into CHC coated neutral serum monovettes (9 mL; Sarstedt, Germany). The first milliliter of blood was discharged. Immediately after taking the blood samples, 2 mL blood were carefully filled into CHC-coated microtubes (2.0 mL; Eppendorf, Germany), using CHC-coated pipette-tips (Greiner Bio-One, Germany). Each tube was placed on a spinning wheel inside a heating cabinet at 37°C. During the incubation time of 30 to 120 min, the blood samples were continually maintained in motion at approximately 10 rpm.

The same protocol was applied for the MPC-based model, but neutral monovettes were pre-loaded with either 8 μg/mL FPX (Arixtra; Aspen Pharma Trading Limited, Ireland) or 0.8 IE/mL ENOX (Clexane; Sanofi-Aventis, Germany) before blood-drawing. Subsequently, 2 mL blood were pipetted into MPC-coated microtubes.

Where applicable, thrombin (Merck, Germany) was added in different concentrations. To model endotoxemia, 100 ng/mL lipopolysaccharide (LPS) were added. Throughout all experiments, blood handling and pipetting was performed carefully by trained staff, with only short periods of air exposure and suspended motion of the blood samples.

After incubation, samples were either processed for blood gas analysis and/or pipetted into 3.2% or 3.8% citrated tubes (Sarstedt) or Eppendorf tubes with 0.5 M Ethylenediaminetetraacetic acid (EDTA; Sigma-Aldrich, Germany) for further processing (see S1 Fig for a schematic image of the experimental setup).

### Heparin coating

All materials in contact with blood, including monovettes, microtubes and pipette-tips were coated with a double-layer of the CHC (Corline, Sweden), as previously described [15,21]. In brief, the material was preincubated with 5% ammonium persulfate (Sigma-Aldrich) at 60°C. All following steps were performed at room temperature. Incubation with PAV (a proprietary formula of a polyallylamine) for 15 min and CHC for 60 min was repeated once. Between every step, the material was rinsed carefully using distilled water (Fresenius Kabi, Germany). After 15 min of incubation with borate buffer (pH 9) there followed 10 min of incubation with acetic anhydride borate buffer (pH 10.5), rinsing and air drying. During every step, the material was placed on a rotating wheel with a loading volume of 1 mL for microtubes and 5 mL for monovettes. The protocol was performed with appropriate measures to reduce the possibility of bacterial or LPS contamination. Staining of sample materials from every batch with toluidine blue (Sigma-Aldrich) was performed to verify a successful coating process (S2 Fig).

### MPC coating

MPC polymer Lipidure (NOF Corporation, Japan) was solved in ethanol (Merck) to a final concentration of 3% (w/v) and 250 μL solution were filled into microtubes. To spread the coating evenly over the entire surface, tubes were incubated on a spinning wheel for 30 min. Subsequently, the tubes were washed using phosphate-buffered saline (PBS) without calcium or magnesium (Life Technologies Limited, UK) and dried at 60°C for one hour. The entire procedure was repeated once to provide a complete coating, which was verified by staining of sample materials from every batch with rhodamine (Sigma-Aldrich) (S2 Fig). The protocol was performed under appropriate conditions to reduce the possibility of bacterial or lipopolysaccharide contamination.

### Blood count, laboratory parameters of hemostasis and ELISA

Following the respective incubation, citrate- and EDTA-anticoagulated blood was analyzed by the clinical routine laboratory of the medical center of Ulm for small blood counts (Coulter DxH 800; Beckman Coulter, USA), coagulation parameters (partial thromboplastin time, PTT; international normalized ratio, INR; turbidimetry, Siemens-BCS XP^®^), d-dimers (immunoturbidimetry; Siemens-BCS XP^®^) and platelet function assays (PFA; PFA-200^®^, Siemens). Additionally, blood gas analysis (ABL flex 800; Radiometer Medical ApS, Germany) and rotational thromboelastometry (ROTEM delta, Tem Innovations GmbH, Germany) were performed.

For some experiments, thrombocyte counts were performed manually as indicated in the respective parts of the manuscript and figure legends. For this purpose, 5 μL EDTA blood was mixed with 95 μL thrombocount reagent (Servoprax, Germany). After 15 to 20 min, 10 μL of the mixture was pipetted on each side of the counting chamber and sedimented for 15 to 20 min.

For enzyme-linked immunosorbent assays (ELISA), EDTA was added directly to respective samples after ex vivo incubation as described above. Subsequently, blood samples were centrifuged for 5 min at 800 *g* and 2 min at 16,000 *g*. The following ELISA kits were used, strictly adhering to the manufacturers’ introductions: BD OptEIA Set Human IL-6 ELISA (BD Biosiences, Germany), Human IL-8/CXCL8 DuoSet ELISA (R&D systems, USA), sMAC/TCC ELISA (BD Biosiences, Germany), Human MMP-9 DuoSet ELISA (R&D systems, USA), Human Thrombin-Antithrombin Complex ELISA Kit (TAT) (Abcam, UK).

### ROTEM

For ROTEM analysis, 300 μL citrated blood of each respective specimen was used. Blood directly processed after drawing served as controls. To compare the impact of FPX and ENOX, 100 μL blood were filled in 3.2% citrate tubes after 30 min incubation in the MPC model with either FPX or ENOX. Clotting time (CT), clot formation time (CFT) and maximum clot firmness (MCF), as well as the alpha angle (AA) and A10 were determined for the extrinsic (via EXTEM) and intrinsic systems (via INTEM).

### Flowcytometry and ADP-induced platelet activation

Blood samples (with either FPX or ENOX) were prepared and incubated as described above. At baseline or after ex vivo incubation, 180 μL blood were transferred immediately into 20 μL citrate (0.106 M; from S-Monovette; Sarstedt) and carefully inverted. Citrated blood was diluted 1:12.5 in pre-warmed HBSS^-/-^ (Hanks’ balanced salt solution, no calcium, no magnesium; Gibco Thermo Fisher, Germany) buffer and 40 μL of the diluted samples were mixed with 0.1 mM adenosine diphosphate (ADP) diluted in PBS (Gibco Thermo Fisher) to obtain a final concentration of 5 μM or with PBS only as a control. Following 10 min of incubation at 37°C in a water bath, anti-CD41 (Biolegend, USA) and anti-CD62P (Biolegend) antibodies were added at a 1:100 dilution. Following an additional 5 min of incubation, samples were transferred into 2 mL cold HBSS^-/-^, inverted and again 200 μL were transferred into test tubes for flowcytometry containing 400 μL HBSS^-/-^ for final measurement.

### Statistical analysis

For statistical testing and graphical depiction, Prism9 (GraphPad Software, USA) was used. Descriptive statistics were performed for each data set prior to statistical testing. Data sets were normally assumed to be parametric; the decision to reject parametric distribution was based on rational consideration, and respective data sets are marked accordingly. Outlier testing was performed using the ROUT method prior to statistical testing based on rational decision, with reference to excluded outliers in the figure legends of the respective data sets. If not stated otherwise, data sets are depicted as mean ± standard error of the mean (s.e.m.) to account for the experimental variances of group means, which were tested for statistical significance.

Data sets with parametric distribution were either tested for statistical significance using the t-test (two experimental groups) or repeated measures (RM) one-way ANOVA/Prism 9 mixed-effects model (more than two experimental groups). Because sphericity could not be assumed, the Geisser-Greenhouse correction was applied. Data sets with non-parametric distribution were tested using either the Friedman or Kruskal-Wallis test with Dunn’s correction. Post-hoc tests with correction for multiple comparisons were used as described in the respective sections and figure legends. Results were considered statistically significant at p-values <0.05 and (for reasons of simplicity) the level of significance was depicted as follows: *p<0.05; **p<0.01; *** p<0.001; **** p<0.0001; ns: not significant.

## Results

### CHC impairs hemostasis, while routine parameters of coagulation remain stable throughout incubation with either ENOX or FPX

**Fig 1:**
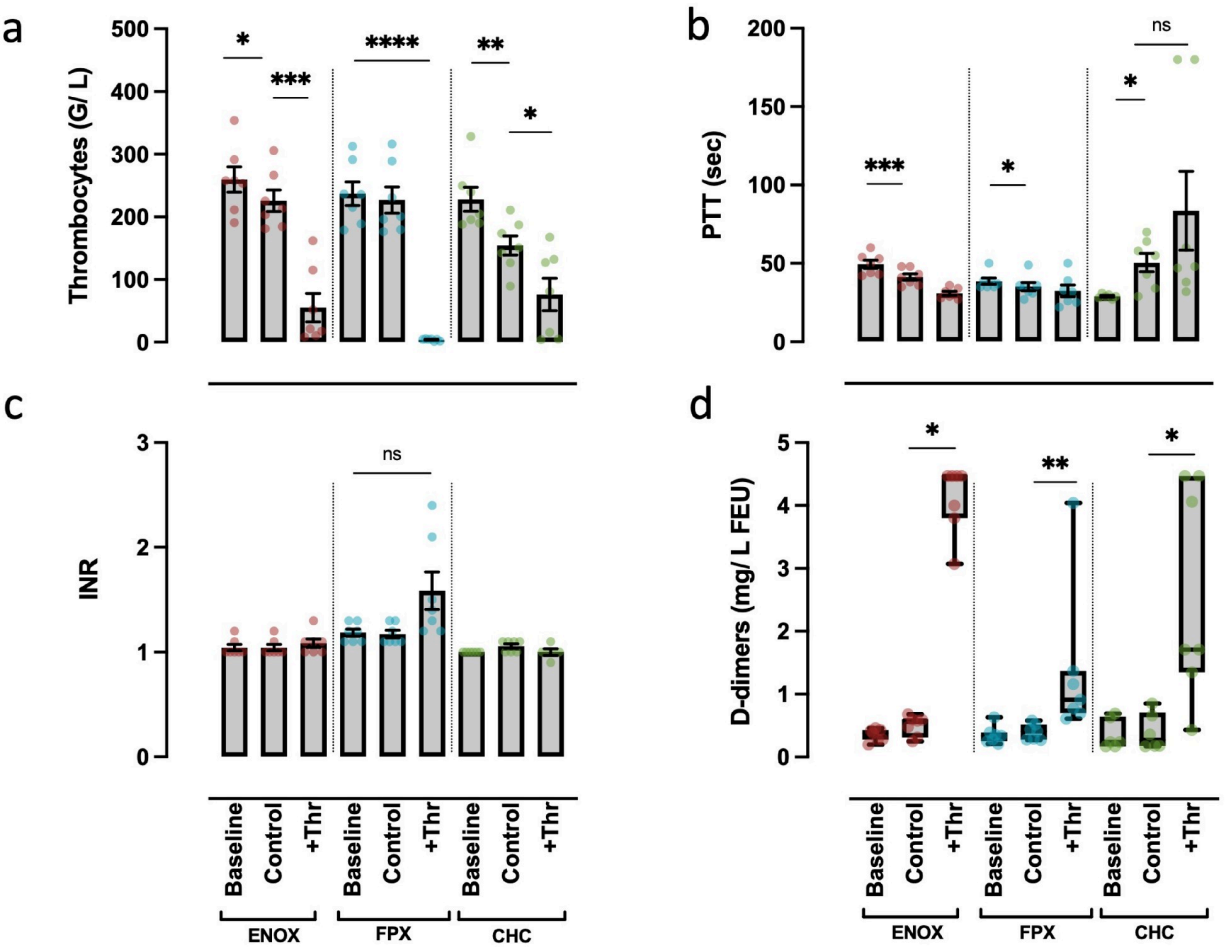
Laboratory parameters of plasmatic and cellular coagulation in three different thrombin-activated models. Thrombocyte counts (a), PTT (b), INR (c) and d-dimers (d) were measured. For each experiment, samples were taken both directly after blood-drawing (Baseline) and after 30 min of incubation without (Control) and with 1 U/mL Thrombin (+Thr). Each experiment was performed with either CHC coating or MPC coating plus anticoagulation with either enoxaparin (ENOX) or fondaparinux (FPX). For statistical analysis, each group (FPX, ENOX, CHC) was tested separately. RM one-way ANOVA with Geisser-Greenhouse correction with Dunnett’s post-hoc test (a-c) and Kruskal-Wallis test with Dunn’s correction (d) were performed. *p<0.05; **p<0.01; *** p<0.001; **** p<0.0001; ns: Not significant. Values presented as mean ± s.e.m (a, b, c) and median ± range (d). All samples: n = 7, except for CHC baseline (b, c), CHC Control (d): n = 6; CHC +Thr (c): n = 5, due to technical reasons (b, c) and exclusion of an outlier (d: 3.37 mg/ L FEU).

To evaluate to what extent an initiation of coagulation is possible despite anticoagulation, we added thrombin as a strong activator of the coagulation system to serve as a positive control. As an early and clinically established surrogate parameter for cellular activation of the coagulation cascade, we determined the platelet count both directly after blood-drawing and after 30 min of incubation with and without 1 U/mL thrombin. In the CHC and the MPC/ENOX models, but not in the MPC/FPX model, a small but significant decrease in the platelet count occurred in control samples (Fig 1a). In all models, a significant reduction in the platelet count was evident after thrombin addition, being most pronounced with the FPX protocol (Fig 1a). When using a protocol without prior MPC coating, solely adding FPX or ENOX, thrombocyte counts decreased slightly already after 30 min (with a statistically significant difference when using ENOX as an anticoagulant). By contrast, when incubating whole blood in MPC-precoated tubes without the addition of an anticoagulant, platelet counts were significantly reduced due to pronounced activation of the coagulation cascade and thrombus formation (S3a Fig). As expected, matrix metalloproteinase 9 (MMP9) as a parameter of immune cell activation was markedly increased after incubation in MPC coated tubes without additional anticoagulants. No differences between the anticoagulants could were observed regarding MMP9 (S3b Fig).

The effect of thrombin on thrombocyte activation and consumption was dose dependent to some extent (S4 Fig). However, there was no relevant increase of effect with either protocol when stimulating with 2 U/mL compared to 1 U/mL. Of note, the maximum achievable effect was more pronounced with FPX (with extensive clot formation), whereas with ENOX, platelet counts reached a mean nadir of approximately 100 G/L after incubation with 1 U/mL thrombin, with no relevant changes after further dosage increases.

Only incubation of whole blood in CHC-coated tubes for 30 min prolonged PTT, with two samples showing PTT values above the detection limit (Fig 1b). Using FPX, the PTT remained unchanged irrespective of thrombin. The INR values did not change on incubation using either of the protocols (Fig 1c). Addition of thrombin did not alter the INR group means, neither in the MPC/ENOX nor in the CHC model. By contrast, higher INR values were obtained after adding thrombin in the FPX protocol, although the difference failed to reach statistical significance (Fig 1c). These changes did not appear to be dose dependent in any relevant manner, although PTT shortening was more pronounced with higher thrombin concentrations (S4c Fig).

D-dimers, as degradation products of fibrinolysis, were barely detected in the incubation controls of any control condition (Fig 1d). By contrast, after addition of thrombin, a robust and statistically significant increase of d-dimers was found in each model (Fig 1d).

### Extended incubation periods led to increased fibrinolysis and reduced thrombocyte counts using the heparin coating, whereas coagulation parameters remained stable with ENOX and FPX

Blood was incubated up to 120 min to assess the integrity of cellular and plasmatic coagulation and background activation of innate immunity as well as changes of blood gases and the acid-base balance over the course of time.

**Fig 2:**
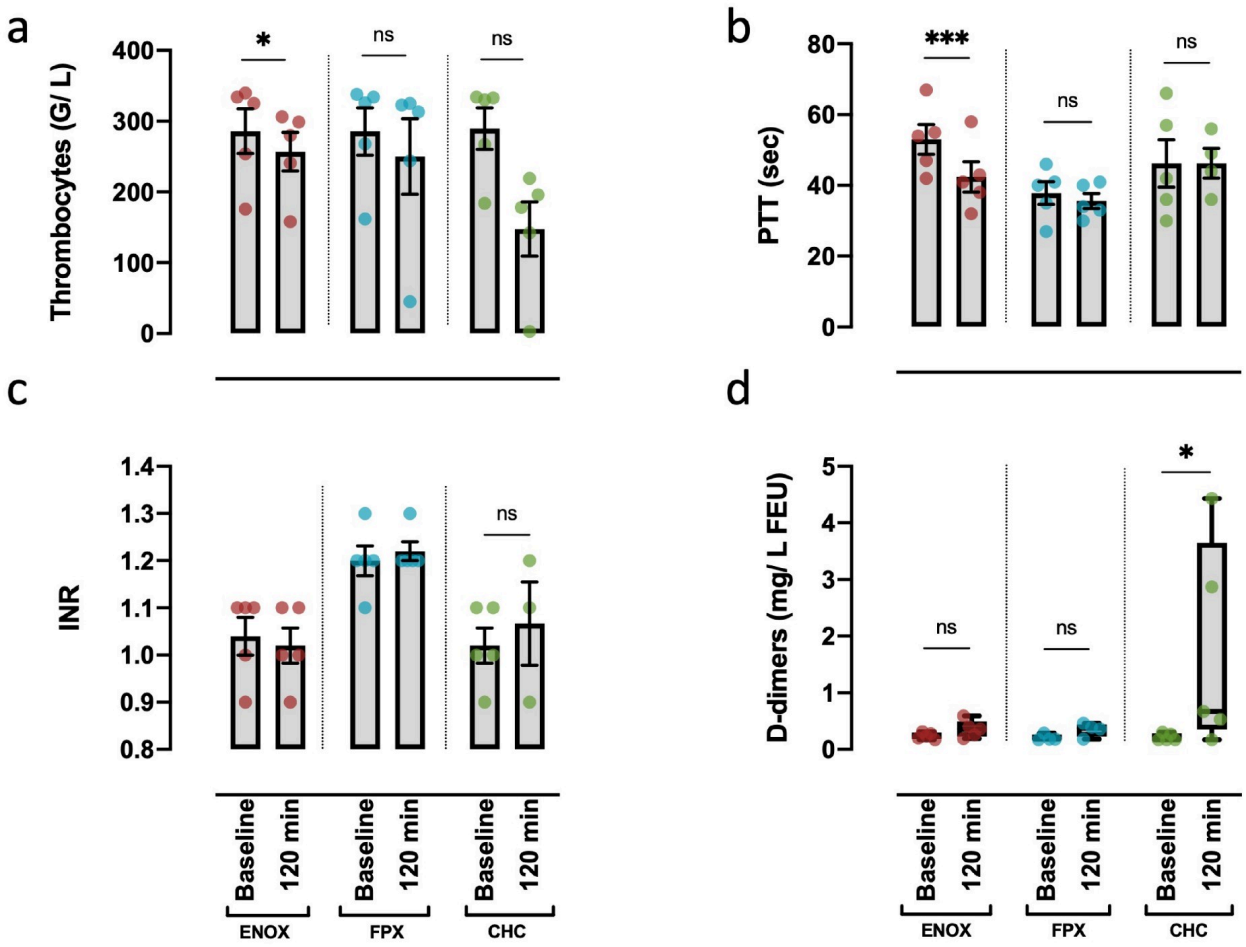
Differential impact of various anticoagulant strategies on coagulation parameters two hours after incubation. Measurement of the global parameters thrombocyte count (a), PTT (b) and INR (c) for cellular and plasmatic coagulation. D-dimers were measured as parameter for fibrinolysis (d). Values were obtained by measuring blood samples both directly after blood drawing (Baseline) and after 120 minutes of incubation. For statistical analysis, RM one-way ANOVA (a, c) and mixed-effect model (b) were performed with Geisser-Greenhouse correction. For each model mean values at baseline and 120 min were compared using Sidaks multiple comparison test. For d-dimers the non-parametrical Kruskal-wallis test (d) was performed, comparing mean values at baseline and 120 min of each model using Dunn’s multiple comparison test. *p<0.05; *** p<0.001; ns: Not significant. Values presented as mean + s.e.m (a, b, c) and median ± range (d). All samples: n = 5, except for CHC 120 min (b, c), FPX baseline, 120 min (d): n = 3–4, due to technical reasons (b, c) and due to exclusion of an outlier (d: 4.43 mg/L FEU).

In the MPC/ENOX protocol, a slight, yet statistically significant decrease in thrombocyte counts after 120 min was obtained (Fig 2a). An even more pronounced, however not statistically significant difference of group means was observed using the CHC protocol (Fig 2a). A moderate decrease of leukocytes and a non-significant increase in Interleukin 6 (IL-6) could be observed in all three approaches after 120 min, which was most pronounced with CHC (S5a and S5c Fig). Similarly, levels of the soluble membrane attack complex (sMAC), as a surrogate for complement activation, were increased after 120 min, which reached statistical significance with CHC (S5d Fig).

The PTT was not significantly altered after 120 min in the intergroup comparison (Fig 2b). By contrast, a statistically significant reduction of the PTT after 120 min compared to baseline was detected with ENOX. This alteration appeared to be dose dependent to a limited extent (S4c Fig). Unexpectedly, baseline values already differed among experimental groups, reaching unphysiological levels in both the MPC/ENOX and CHC groups.

In the MPC-based protocols the INR values remained unchanged after 120 min (Fig 2c). In the CHC model, a slight but non-significant increase was observed after incubating for 120 min. Moreover, in the MPC-based protocols, no increase in d-dimers could be detected after 120 min (Fig 2d). One sample of the FPX group was excluded due to unphysiologically high values, that remained unchanged after incubation and were identified as outliers by the ROUT test (Q = 1%). By contrast, when using the CHC-coating a significant increase in fibrin-degradation products was observed (Fig 2d). As an additional marker of activated coagulation, thrombin-antithrombin complexes (TAT-complexes) showed a slight, yet not significant increase with either protocol (S5e Fig).

### Blood gas alterations showed a similar time-dependency in all protocols

**Fig 3:**
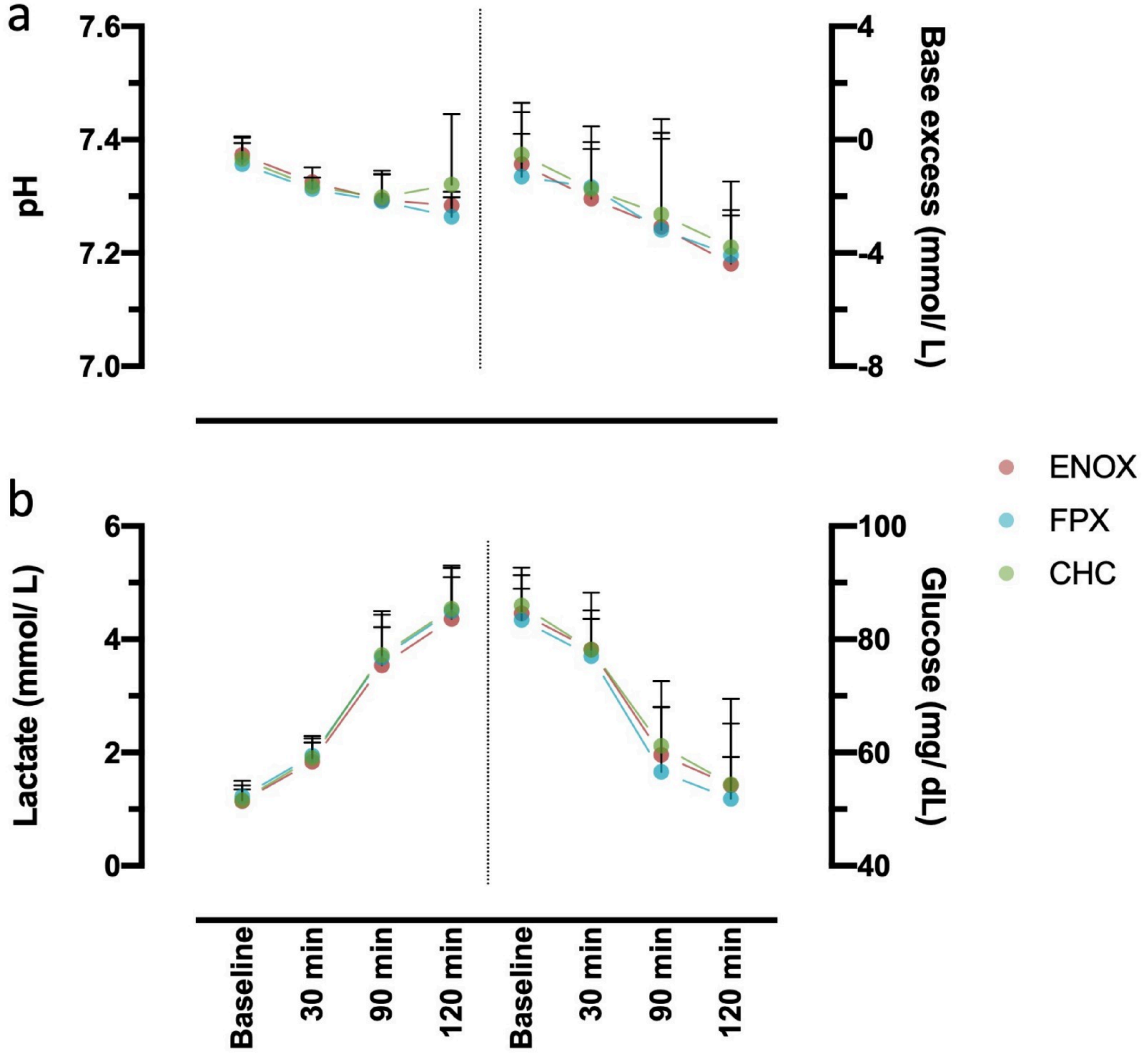
Comparison of the acid-base status and glucose metabolism after 30, 90, 120 minutes of incubation. Blood samples were incubated for 30, 90 or 120 minutes at 37°C using the MPC-based coating protocol with either ENOX or FPX as anticoagulant or the CHC-coating protocol. Base excess and pH (a) as well as lactate and glucose (b) were measured via blood gas analysis. No differences among the models were observed at any of the investigated timepoints. Data was considered exploratory and for further reference, therefore no statistical testing was performed. Values are presented as mean + 95% CI. All samples: n = 5.

The acid-base status of each sample was obtained via blood gas analysis both directly after blood drawing and after incubation for the respective time periods. There was a slight decrease of the blood pH in a time-dependent manner in each of the three models (Fig 3a). The base excess decreased only slightly in all three models (Fig 3a). In all three models, lactate levels displayed the same increasing pattern associated with a decrease in glucose (Fig 3b). Neither relevant changes over time nor differences between the protocols were detectable regarding the blood electrolytes K^+^, Ca^2+^ or Na^+^ or the blood gases pCO_2_ and pO_2_ (S1 and S2 Tables).

### Thromboelastographic analyses remained unaltered by incubation either with FPX or ENOX

To further analyze the differential impact of either ENOX or FPX as an anticoagulant for extracorporeal incubation of human whole blood, we employed ROTEM analysis, PFA and flowcytometry.

The extrinsic system measured by EXTEM showed no significant changes in the experimental system regarding CT or CFT with either protocol (Table 1). However, ENOX showed a more pronounced impact on the intrinsic system, than FPX, regarding the INTEM-CT. Regarding the INTEM-CFT, -MCF and clot firmness (A10), neither FPX nor ENOX resulted in a relevant prolongation (Table 1).

**Table 1:** Changes in ROTEM due to incubation with fondaparinux or enoxaparin.

	Parameter	Groups			ANOVA
		Baseline	ENOX	FPX	
INTEM					
	**CT** [sec]	194.00 (± 5.1)	236.70 (± 11.8)	197.50 (± 8.4)	n.s.
	**CFT** [sec]	83.86 (± 5.0)	92.67 (± 15.5)	97.50 (± 18.3)	n.s.
	**MCF** [mm]	62.43 (± 1.6)	58.00 (± 4.0)	61.75 (± 1.7)	n.s.
EXTEM					
	**CT** [sec]	71.29 (± 2.9)	61.33 (± 5.0)	87.75 (± 10.0)	n.s.
	**CFT** [sec]	88.29 (± 7.3)	76.33 (± 12.2)	104.80 (± 14.2)	n.s.
	**MCF** [mm]	65.29 (± 1.9)	63.33 (± 3.3)	62.50 (± 2.6)	n.s.
	**AA** [°]	73.86 (± 1.7)	75.33 (± 2.8)	70.00 (± 2.5)	n.s.
	**A10** [mm]	57.86 (± 2.4)	56.33 (± 4.4)	53.50 (± 3.9)	n.s.

ROTEM-analyses were done with freshly drawn blood (Baseline) and after 30 minutes of incubation in the MPC model using enoxaparin (ENOX) or fondaparinux (FPX). Clotting time (CT), clot formation time (CFT) and maximum clot firmness (MCF) were measured for the extrinsic and intrinsic systems. α-angle (AA) and amplitude after 10 min (A10), showing characteristics of the formed thrombus, were measured in EXTEM.

For statistical analysis, one-way ANOVA (Brown-Forsythe and Welch) with Dunnett´s post-hoc test with adjustment for multiple comparisons was performed. Values as mean ± s.e.m. Differences in sample size due to experimental design: Baseline: n = 7; FPX: n = 4; ENOX: n = 3.

### Platelet function was preserved in blood either anticoagulated with ENOX or FPX

**Fig 4:**
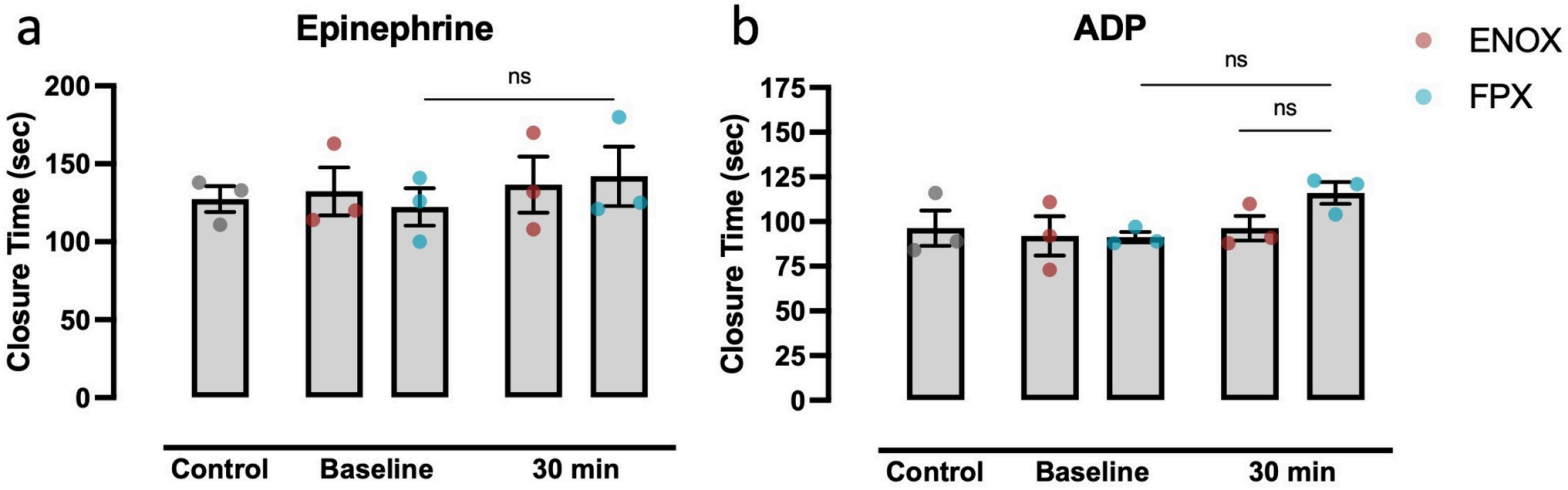
Preservation of platelet function at baseline and after incubation for 30 minutes. Measurements of thrombocyte function and initial platelet-thrombus formation were performed employing a routinely used platelet function assay (PFA). Therefore, anticoagulated blood was stimulated with epinephrine (a) or ADP (b) on a collagen-coated surface. For control values, freshly drawn blood was only anticoagulated with 3.8% citrate. PFA was also performed with citrated blood plus either fondaparinux (FPX) or enoxaparin (ENOX), both freshly drawn (Baseline) and after 30 minutes of incubation (30 min) in MPC-coated tubes. For statistical analysis, RM one-way ANOVA with Geisser-Greenhouse correction was performed, using Tukey’s post-hoc testing with adjustment for multiple comparisons. Values are presented as mean ± s.e.m. All samples: n = 3.

Platelet function was determined via PFA. Blood anticoagulated with FPX or ENOX showed no significant change in the closure time after stimulation with either ADP (Fig 4a) or epinephrine (Fig 4b), compared with citrated blood both at baseline and after 30 min (Fig 4a and 4b).

**Fig 5:**
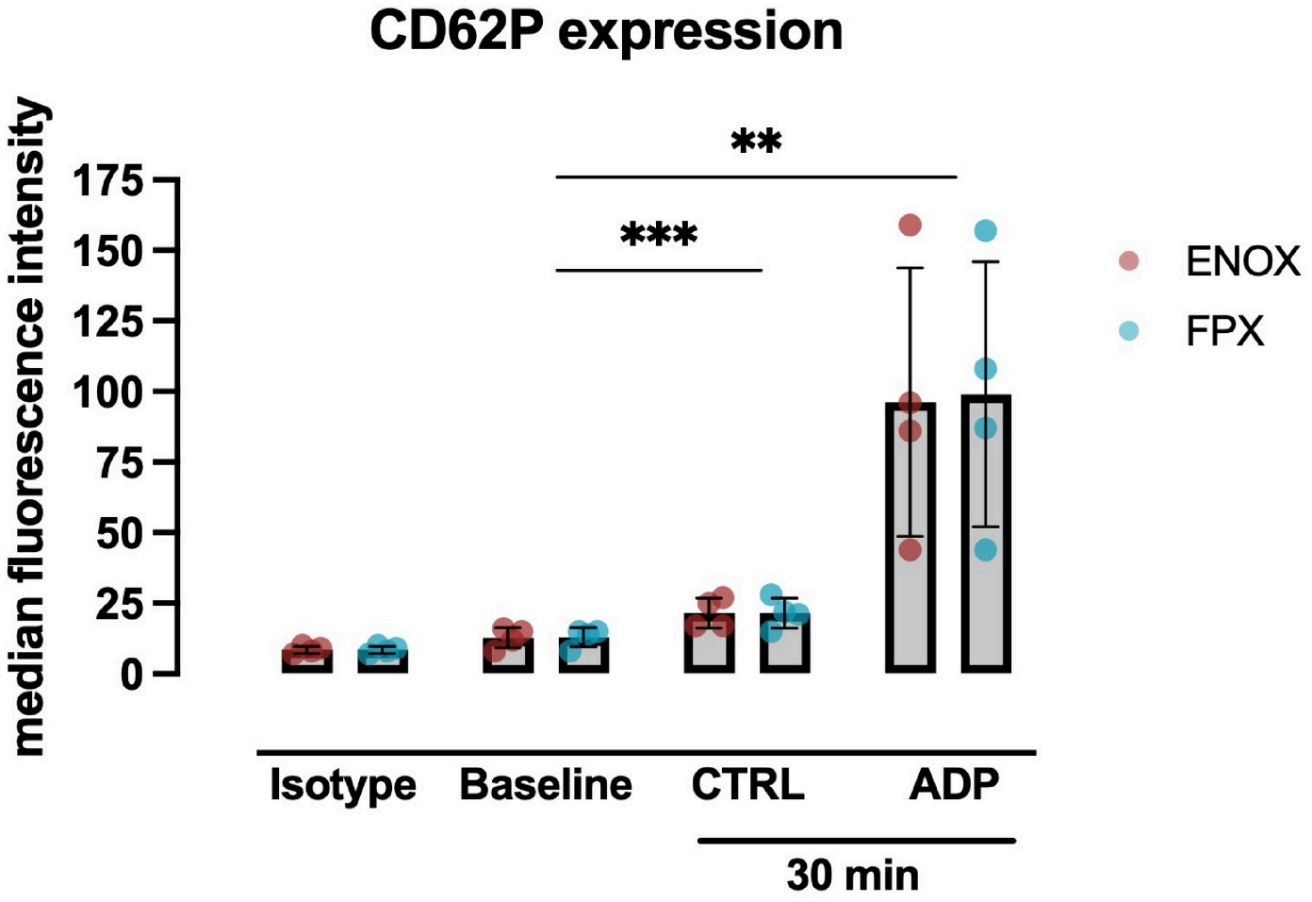
Platelet activation marker expression after ADP-stimulation. Flow-cytometric analysis of whole blood samples was performed using p-selectin (CD62P) as an activation marker on thrombocytes. Freshly drawn blood (Baseline) was compared with samples after 30 minutes of incubation (30 min) and after additional stimulation with ADP (ADP). Either enoxaparin (ENOX) or fondaparinux (FPX) was used as an anticoagulant in MPC-coated tubes. For statistical analysis, RM two-way ANOVA with Geisser-Greenhouse correction was performed using Tukey’s post-hoc testing (Isotype values were excluded from testing). **p<0.01; *** p<0.001. Values are presented as mean ± s.e.m. All samples: n = 4.

Flowcytometric analyses of whole blood samples were performed determining p-selectin (CD62P) as an activation marker on thrombocytes. CD62P expression of freshly drawn blood was compared with samples after 30 min of incubation and after stimulation with ADP. The mean difference in CD62P expression between freshly drawn blood and after incubation for 30 min was statistically significant, but only marginally and, therefore, might have been a statistical artefact due to small variances (Fig 5). By contrast, anticoagulation with either ENOX or FPX did not impair the potential of platelets to be activated, because stimulation with ADP induced a strong increase of CD62P.

### Modeling endotoxemia induced a significant innate immune system activation without relevant coagulation system activation

To assess the integrity and responsiveness of immune cells and the usability of the MPC-based protocols to simulate endotoxemia ex vivo, whole blood anticoagulated with either ENOX or FPX was incubated for 30 min under stimulation with 100 ng/mL LPS with or without additional 0.5 U/mL thrombin.

**Fig 6:**
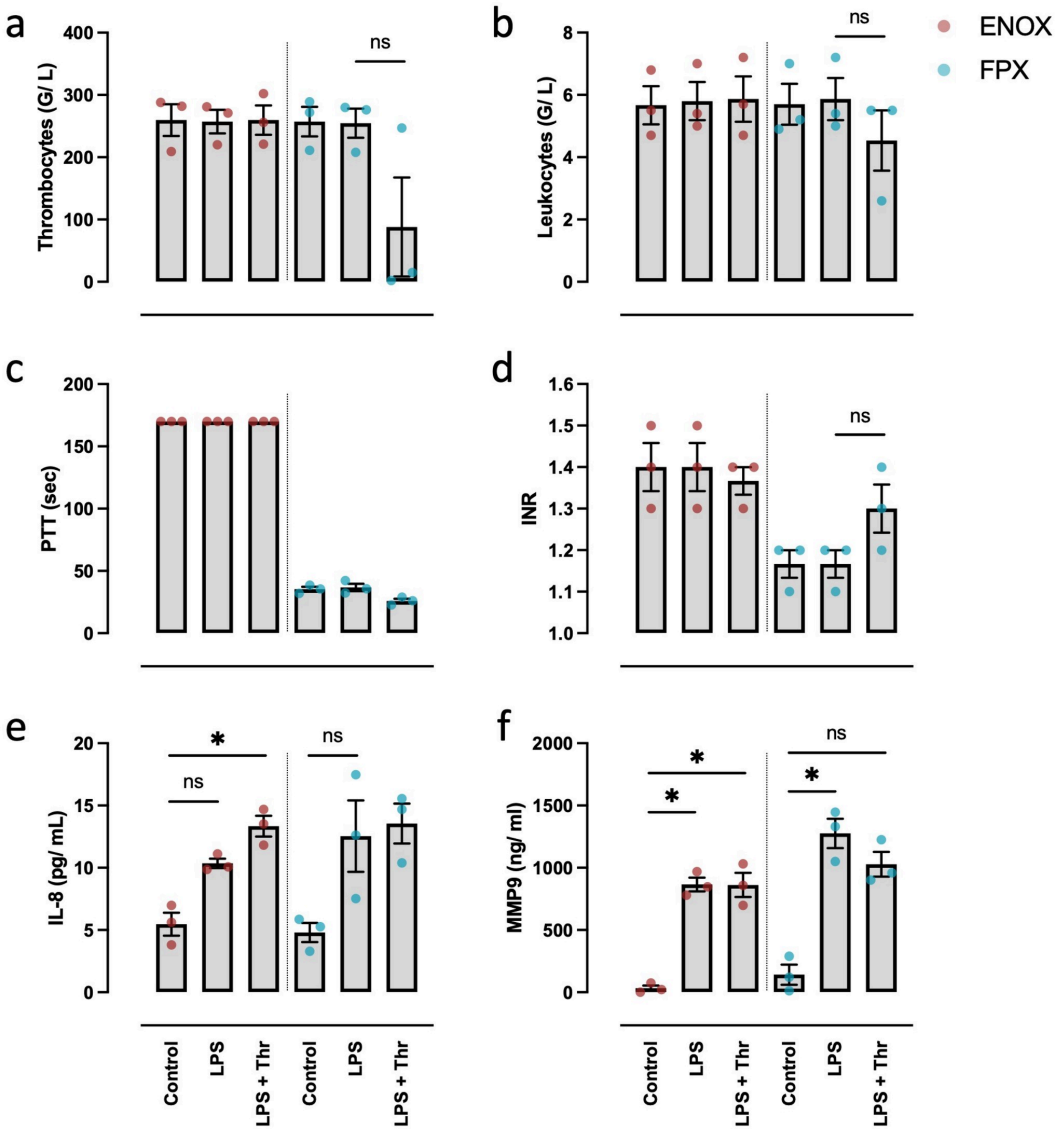
Effects of LPS stimulation with and without additional thrombin-driven activation of the coagulation system. Human whole blood was incubated for 30 min with either PBS (Control) or 100 ng/mL LPS plus or minus 0.5 U/mL thrombin (LPS and LPS + Thr, respectively). Thrombocyte and leukocyte counts (a,b) showed no significant changes with either stimulant, except for a slight decrease of both platelets and leukocytes when stimulated with both LPS and thrombin. The PTT remained unchanged when using the FPX protocol (c). The PTT values of the ENOX group cannot be interpreted, because they were artificially prolonged beyond the detection limit in all samples. An obtainable, yet statistically insignificant increase of the INR was observed when incubating whole blood with both LPS and thrombin using the FPX protocol (d). Both IL-8 and MMP9 were elevated after incubation with LPS in either model (e, f). Thrombin did not show a significant additional effect. For statistical analysis, RM two-way ANOVA with Geisser-Greenhouse correction was performed using Tukey’s post-hoc testing for each group (FPX and ENOX) separately. *p<0.05. Values are presented as mean ± s.e.m. All samples: n = 3.

After this time-period, LPS alone did not cause relevant activation of the coagulation system as obtained by platelet counts and INR (Fig 6a and 6d). Under additional stimulation with 0.5 U/mL thrombin, samples anticoagulated with FPX, but not with ENOX, displayed a decreased platelet count and an increased INR. Regrettably, PTT changes could not be assessed in this experiment, as samples of the ENOX group appeared to have been compromised either post-incubation or during laboratory analysis, with all values were prolonged beyond the upper detection limit (Fig 6c).

Both with the ENOX and FPX protocols, IL-8 and MMP9 as surrogate parameters of activation of immune cells were significantly increased. Thrombin addition did not show a relevant additional effect (Fig 6e and 6f). Overall leucocyte counts differed only after the combined stimulation with both LPS and thrombin when employing the FPX protocol (Fig 6b).

## Discussion

In the present study we compared different protocols for an ex vivo incubation of human whole blood regarding their impact on plasmatic and cellular hemostasis by determining thrombocyte counts and global parameters of coagulation. Following our initial findings, we further analyzed the impact of ENOX and FPX as anticoagulants, respectively, on platelet and hemostatic function.

No relevant changes regarding basic coagulation parameters were observed after ex vivo incubation of whole blood anticoagulated with either FPX or ENOX. Therefore, marked activation of the cellular and/or plasmatic compartments of hemostasis appears unlikely. However, a minor difference in platelet counts after 30 min of incubation using ENOX as an anticoagulant was statistically significant, but most likely without clinical relevance or time-dependency.

Because FPX and ENOX show no relevant impact on the PTT in vivo, this parameter is not routinely used as a laboratory marker to monitor treatment [19]. By contrast, in our experiment, the PTT was significantly decreased with ENOX, which was further enhanced by the addition of thrombin and was dose dependent to some extent. Whether this correlates to an artefact in the laboratory analysis (e.g. through interference of the remaining active thrombin in the analyzed sample) and may have any translational relevance needs to be further evaluated.

The strong decrease of platelets observed when using the CHC protocol could either be due to an aggregation of thrombocytes through an activation of the coagulation system or a result of cell adhesion on the surface of the tube. Previous work showed that two layers of UFH, as used in our protocol, led to less cell activation compared to a single layer [15]. However, it has also been described, that proteins like fibronectin, fibrinogen and vWF still adhere to a UFH-coated surface [22]. There have been numerous recent studies, investigating surface activation of both the coagulation cascade and the complement system. For example, with the CHC-coating less of the activated form of factor XII is adsorbed on the surface in contact with blood compared to an uncoated surface [23]. Similarly, due to the molecular structure of MPC a reduced adsorption of proteins, such as high-molecular-weight kininogen and factor XII, occurred [11]. A prolonged PTT reflects the heparin-effect on human blood [24]. Because no additional heparin was added to the freshly drawn blood, the observed PTT prolongation could either reflect a shedding of the CHC coating into the blood upon incubation or a consumption of coagulation factors upon clotting. The lesser staining with toluidine blue after 30 min incubation with blood is supportive of the theory of heparin shedding (cf. S2 Fig). However, PTT measurements after 120 min failed to reproduce the above-mentioned effect. This contradictory finding would require further characterization when evaluating CHC-based coating protocols for clinical use.

D-dimers, as established indicators for thrombus formation [25], remained unchanged in each of the used protocols, thus any relevant activation of the coagulation cascade appears unlikely. However, in the CHC-coated tubes a significant increase in d-dimer plasma concentrations, as well as an increase of TAT-complexes was observed after extended incubation, which, in conjunction with a decrease in thrombocyte counts, may reflect an ongoing amplificated activation of the coagulation system that was not yet apparent after 30 min.

By thrombin addition, we evaluated whether thrombocytes and the coagulation cascade could still be activated despite anticoagulation and whether there were differences between the employed protocols. Platelet counts decreased drastically in all models after activation with thrombin and was most pronounced with FPX. These effects were dose dependent, showing the integrity of a graduated response of the coagulation system to activation. In the CHC model, the prothrombin time was further prolonged while the INR values remained unchanged after adding thrombin, which might reflect the start of coagulation component consumption. However, results in the CHC model were less reproducible, as indicated by the broad distribution of experimental values. This lack of reliability of the UFH coating is a phenomenon also observed in patients who receive systemic UFH, because its effect was reported to be less predictable and controllable [26]. Regarding the above-mentioned hypothesis of heparin-shedding from the coating layers, this may reflect an inconsistency in the extent of heparinization of the whole blood samples. However, data considering the release of heparin from the coated surface into the blood remains inconclusive [21]. By contrast, it has been conclusively demonstrated that coatings with MPC remain unchanged on the grafts´ surface in vitro [27] and in vivo for at least 6 months [28]. This was also confirmed with the rhodamine staining, which showed macroscopically consistent staining before and after 30 min incubation of whole blood (S2 Fig). In the clinical setting, a possible shedding might be negligible, because systemic heparinization is applied. To limit the impact on the coagulation system, we decided not to add heparin, especially as comparable protocols using additional heparin showed a considerable impact on hemostasis [29].

To further address the scientific and clinical applicability of the protocols, we also investigated basic physiological parameters by blood gas analyses. As expected for a closed system, plasma glucose concentrations diminished time-dependently due to consumption by the ongoing (anaerobic) cellular metabolism. Correspondingly, lactate levels increased, inducing a mild metabolic acidosis after 90 min. These findings reflect a noticeable limitation of our study and the models presented. Our observations of a developing metabolic shift towards acidosis via the accumulation of lactate are in accordance with findings from a comparable study working with human whole blood [29]. Even so, within the first 30 min of ex vivo modeling, these parameters appeared constant and thus the protocols may still be useful for valid experimental simulation of in vivo processes. Moreover, regarding the parameters investigated in this study, these changes did not seem to affect the hemostatic qualities of the blood and did not lead to any relevant hemolysis. However, it should be noted that an extended incubation led to a relevant activation of the complement system, which may at least in part be enhanced through lactate accumulation, as described previously [30]. Additionally, acidosis is known to affect both platelet function and plasmatic hemostasis and impairs immunological functions [31,32]. Using the MPC-based protocols, platelet function remained unimpaired after 30 min, when pH and lactate levels were still within physiological ranges. Whether longer incubation times in a closed system would affect the presented read-outs is of limited clinical relevance and was beyond the scope of this study.

The CHC protocol presented here had a series of limitations compared to the MPC-based protocols, which question its usability for further scientific investigations regarding hemostasis. The MPC coating itself has no antithrombogenic activity and only acts as antifouling agent [10], such that the addition of anticoagulants was necessary. Of note, in our experiments, the release of pro-inflammatory MMP9 was slightly higher in MPC-coated than in -uncoated tubes, a finding that necessitates confirmation in future studies and, if confirmed, further investigation regarding the underlying immunobiological mechanisms. Because both protocols investigated in the context of MPC-coated tubing performed equally well, we then designed subsequent experiments to further characterize and differentiate the impact of both anticoagulants on platelet function and physiological clot formation.

Recently, point-of-care thromboelastometry has become more established in clinical use [33], but data on the impact of FPX and ENOX on ROTEM parameters are very limited. In our study, no statistically significant impact of either anticoagulant was detectable. The minimal changes in the CT with MPC/ENOX was comparable to previous results, where a dose-dependency on the prolongation of the CT could be determined [34]. In an animal model, FPX led to an increased CT an CFT in INTEM [35]. In support of our results, a prolonged INTEM-CT at a high FPX concentration was shown previously [36] and the MCF was not impaired in any of the previous studies [34,35,37,38].

Patients undergoing extracorporeal circulation frequently display impaired platelet function [39,40]. Whether different anticoagulants may contribute to or even ameliorate this effect remains unclear. To characterize platelet functionality in the presented protocols, we used flowcytometry and PFA to quantify background platelet activation and preservation of reactivity. Because no relevant increase in CD62P expression on isolated platelets was detected, relevant artificial activation of platelets during the preparation and incubation of the blood samples or through surface interaction with the MPC seems unlikely. Moreover, both anticoagulants did not appear to compromise either the initial thrombus formation through platelet adhesion or activation nor platelet activation by ADP. In accordance, previous investigations with fondaparinux, showed no difference between high and low dosage in either PFA or Multiplate analysis [36]. However, previous studies showed that, platelets treated with ENOX expressed less CD62P after ADP stimulation compared to those treated with UFH [41] and showed a reduced capacity to bind fibrinogen [42], suggesting an inhibitory effect of ENOX on platelet reactivity.

Most vitro models using human whole blood function by recalcification of citrated blood. However, it has been determined that some effects of citrate on blood components cannot be reversed by adding calcium [43]. Therefore, an increased response of thrombocytes after stimulation with ADP could be observed in citrated blood compared to other anticoagulants like heparin, hirudin and enoxaparin [44]. Scientific models for incubating human whole blood ex vivo to address immunological and pharmacotherapeutic questions have been applied successfully [29,45–47]. However, these models have limitations in investigating coagulation physiology, as they relied on substantial anticoagulation. Therefore, a model of largely unimpaired hemostasis may prove useful, either for simulating an inflammatory environment or to analyze potential pharmacodynamic effects on hemostasis.

The use of extracorporeal oxygenation to treat patients with severe sepsis is currently under clinical investigation [48]. Additionally, patients undergoing ECMO are at a higher risk to develop sepsis [49]. To further investigate the integrity of the immune system in the MPC-based models and the applicability of these models for scientific use, we modeled endotoxemia by adding LPS to the incubated whole blood. Within 30 min, a significant release of pro-inflammatory cytokines was observable in both models. Interestingly, this pro-inflammatory response to LPS stimulation was insufficient to cause relevant changes in platelet counts or routine parameters of coagulation. Adding thrombin had only the expected minor effect on these parameters without a potentiation of the impact through LPS.

When considering the presented data, based on their more predictable and reproducible effects concerning anticoagulation and sustained functionality, both protocols based on MPC coating and additional anticoagulation with LMWH or FPX may yield a better clinical applicability and prove safer for patients undergoing extracorporeal circulation. For translational purposes, future studies are necessary to fully characterize the benefits and limitations of each individual approach, and whether coating with an antifouling substance such as MPC may even be applicable or if different substances or even enhanced materials may be superior in regard to coagulation and inflammation. For experimental purposes, employing an MPC-based protocol with additional anticoagulation, may yield an affordable and easy-to-apply approach to relevantly model coagulation and inflammation processes in human whole blood or to analyze substance dynamics and toxicological effects. In this regard, it seems suggestive to use an FPX-based protocol, because FPX appears less prone to artificial changes of global parameters of coagulation, yielded more stable platelet counts throughout incubation and showed a better-preserved reactivity to pro-coagulatory stimuli.

## Supporting information

S1 Fig Experimental setup for ex vivo incubation of human whole blood. For the ex vivo incubation of human whole blood, blood samples were drawn from the cubital vein into neutral tubes, either coated with MPC and preloaded with FPX or ENOX respectively or coated with CHC (a), then gently pipetted in precoated Eppendorf tubes (b). Activating agents were added as described in the respective sections. Subsequently, the sample tubes were carefully placed on a rotating wheel (c) in an incubator (d) with the temperature set to 37.0°C. Following incubation, samples were processed for further analysis as stated in the respective sections. Image created with BioRender.com.
(EPS)

S2 Fig Staining test for coating and wash-off after incubation. Spot checks for complete staining with toluidine blue (for CHC coating, upper panel) and with rhodamine (for MPC coating, lower panel) before and after incubation with whole blood for 30 minutes. In particular, the CHC coated tubes showed a slight wash-off, as apparent by the fainter staining after incubation.
(EPS)

S3 Fig Comparison of different coating protocols. Blood samples were incubated for 30 min and thrombocytes counts and plasma MMP9 were determined before (Baseline) and after incubation. The different conditions for incubated samples were either using the MPC model without anticoagulation (MPC), or with the addition of anticoagulant fondaparinux (FPX) or enoxaparin (ENOX) to either MPC-coated (+MPC) or uncoated (−MPC)tubes. Incubation for 30 min with ENOX as an anticoagulant caused a slight, but still statistically significant decrease in thrombocyte counts (a), which was even more pronounced when incubating whole blood without prior coating of the material with MPC. Incubating whole blood in MPC-coated tubes omitting additional anticoagulants led to a significant drop in thrombocyte counts due to clot formation (inset). MMP 9 levels were significantly increased after incubation without additional anticoagulants (b). Additionally, there was a slight, yet statistically insignificant increase of MMP9 after incubation with ENOX as an anticoagulant that appeared even more pronounced when whole blood was incubated in MPC coated tubes. For statistical analysis, RM one-way ANOVA (a) and mixed-effect model (b) were performed with Geisser-Greenhouse correction. Sidaks multiple comparison test was used for post-hoc intergroup comparison. Values are presented as mean ± s.e.m; All samples: n = 4, except for MPC (a): n = 3, due to exclusion of an outlier (249 G/L). *p<0.05.
(EPS)

S4 Fig Response to different thrombin concentrations in the MPC model. Blood samples were incubated with either PBS (Control), 0.1 U/mL, 1 U/mL or 2 U/mL thrombin (U Thr) using the MPC model with either fondaparinux (FPX) or enoxaparin (ENOX). Determination of thrombocytes (a), leukocyte count (b), PTT (c) and INR (d) was performed before (Baseline) and after 30 min of incubation. For statistical analysis, RM one-way ANOVA (ENOX group) and mixed-effect model (FPX group) with Geisser-Greenhouse correction were performed using Dunnett’s post-hoc testing (comparing to control values). *p<0.05; **p<0.01; *** p<0.001; ns: Not significant. Values are presented as mean ± s.e.m; All samples: n = 5, except FPX (2 U/mL): n = 4, due to technical reasons.
(EPS)

S5 Fig Changes in blood count, complement activation and plasma IL-6 over time. Blood samples were incubated over 120 min using either the CHC or MPC model with fondaparinux (FPX) or enoxaparin (ENOX). Measurements of leukocytes, hemoglobin (Hb), interleukin 6 (IL-6), sMAC, and thrombin-antithrombin complexes (TAT) were performed before (Baseline) and after incubation. For statistical analysis, RM one-way ANOVA (a) or mixed-effect model (c) with Geisser-Greenhouse correction was performed. For each model mean values at baseline and 120 min were compared using Sidaks multiple comparison test. *p<0.05; ns: Not significant. Values are presented as mean ± s.e.m; All samples: n = 5, except for CHC (d): n = 4, due to technical reasons.
(EPS)

S1 Table Blood electrolytes after 30, 90 and 120 minutes of incubation. Blood samples were incubated using the MPC model with either fondaparinux (FPX) or enoxaparin (ENOX) or with the CHC coating. The blood electrolytes potassium (K^+^), sodium (Na^+^) and calcium (Ca^2+^), measured via blood gas analysis before (Baseline) and after 30, 90, and 120 min of incubation. Data was considered exploratory and for further reference, therefore no statistical testing was performed. Values are presented as mean (± s.e.m). All samples: n = 5, except Na^+^ (90 min): n = 4, due to technical reasons.
(EPS)

S2 Table Blood gases after 30, 90 and 120 minutes of incubation. Blood samples were incubated using the MPC model with either fondaparinux (FPX) or enoxaparin (ENOX) or with the CHC-coating. Measurements via blood gas analysis (BGA) of pO_2_ and pCO_2_ were performed before (Baseline) and after 30, 90, and 120 min of incubation. Data was considered exploratory and for further reference, therefore no statistical testing was performed. Values presented as mean (± s.e.m); All samples: n = 5.
(EPS)

S1 Dataset (XLSX)

S1 File (DOCX)

## Abbreviations

AA: Alpha Angle
ADP: Adenosine Diphosphate
CD62P: Cluster of Differentiation 62p; P-Selectin
CFT: Clot Formation Time
CHC: Corline Heparin Conjugate
CT: Clotting Time
ECMO: Extracorporeal Membrane Oxygenation
ELISA: Enzyme-Linked Immunosorbent Assays
ENOX: Enoxaparin
FPX: Fondaparinux
HBSS: Hanks’ Balanced Salt Solution
IL-6 /IL-8: Interleukin 6/8
INR: International Normalized Ratio
LMWH: Low Molecular Weight Heparin
LPS: Lipopolysaccharide
MMP9: Matrix Metallopeptidase 9
MPC: 2-Methacryloyloxethyl Phosphorylcholine
PAV: Proprietary Formula of a Polyallylamine
PBS: Phosphate-Buffered Saline
PFA: Platelet Function Assay
PTT: Partial Thromboplastin Time
RM: Repeated Measures
ROTEM: Rotational Thromboelastometry
sMAC: Soluble Membrane Attack Complex
UFH: Unfractionated Heparin

## References

[1] Teligui L, Dalmayrac E, Corbeau J-J, Bouquet E, Godon A, Denommé A-S, et al. Ex vivo simulation of cardiopulmonary bypass with human blood for hemocompatibility testing. Perfusion. 2016;31: 376–383. doi: 10.1177/0267659115599454 26243277

[2] Ojeda R, Arias-Guillén M, Gómez M, Vera M, Fontseré N, Rodas L, et al. Study of Biocompatibility of Membranes in Online Hemodiafiltration. Blood Purif. 2020;49: 400–408. doi: 10.1159/000504954 31865336

[3] Roberts TR, Garren MRS, Handa H, Batchinsky AI. Toward an artificial endothelium: Development of blood-compatible surfaces for extracorporeal life support. J Trauma Acute Care Surg. 2020;89: S59–S68. doi: 10.1097/TA.0000000000002700 32251267 PMC7398848

[4] Thomas J, Kostousov V, Teruya J. Bleeding and Thrombotic Complications in the Use of Extracorporeal Membrane Oxygenation. Semin Thromb Hemost. 2018;44: 020–029. doi: 10.1055/s-0037-1606179 28898902

[5] Luyt C-E, Bréchot N, Demondion P, Jovanovic T, Hékimian G, Lebreton G, et al. Brain injury during venovenous extracorporeal membrane oxygenation. Intensive Care Med. 2016;42: 897–907. doi: 10.1007/s00134-016-4318-3 27007107

[6] Krueger K, Schmutz A, Zieger B, Kalbhenn J. Venovenous Extracorporeal Membrane Oxygenation With Prophylactic Subcutaneous Anticoagulation Only: An Observational Study in More Than 60 Patients: THOUGHTS AND PROGRESS. Artif Organs. 2017;41: 186–192. doi: 10.1111/aor.12737 27256966

[7] Wood KL, Ayers B, Gosev I, Kumar N, Melvin AL, Barrus B, et al. Venoarterial-Extracorporeal Membrane Oxygenation Without Routine Systemic Anticoagulation Decreases Adverse Events. The Annals of Thoracic Surgery. 2020;109: 1458–1466. doi: 10.1016/j.athoracsur.2019.08.040 31563493

[8] Gratz J, Pausch A, Schaden E, Baierl A, Jaksch P, Erhart F, et al. Low molecular weight heparin versus unfractioned heparin for anticoagulation during perioperative extracorporeal membrane oxygenation: A single center experience in 102 lung transplant patients. Artif Organs. 2020;44: 638–646. doi: 10.1111/aor.13642 31951030 PMC7317732

[9] Stettler GR, Moore EE, Moore HB, Lawson PJ, Fragoso M, Nunns GR, et al. Thrombelastography indicates limitations of animal models of trauma-induced coagulopathy. J Surg Res. 2017;217: 207–212. doi: 10.1016/j.jss.2017.05.027 28583756 PMC5603369

[10] Asif S, Asawa K, Inoue Y, Ishihara K, Lindell B, Holmgren R, et al. Validation of an MPC Polymer Coating to Attenuate Surface-Induced Crosstalk between the Complement and Coagulation Systems in Whole Blood in In Vitro and In Vivo Models. Macromol Biosci. 2019;19: e1800485. doi: 10.1002/mabi.201800485 30786149

[11] Ishihara K, Aragaki R, Ueda T, Watenabe A, Nakabayashi N. Reduced thrombogenicity of polymers having phospholipid polar groups. Journal of Biomedical Materials Research. 1990;24: 1069–1077. doi: 10.1002/jbm.820240809 2394763

[12] Ishihara K, Oshida H, Endo Y, Ueda T, Watanabe A, Nakabayashi N. Hemocompatibility of human whole blood on polymers with a phospholipid polar group and its mechanism. J Biomed Mater Res. 1992;26: 1543–1552. doi: 10.1002/jbm.820261202 1484061

[13] Feng W, Zhu S, Ishihara K, Brash JL. Adsorption of Fibrinogen and Lysozyme on Silicon Grafted with Poly(2-methacryloyloxyethyl Phosphorylcholine) via Surface-Initiated Atom Transfer Radical Polymerization. Langmuir. 2005;21: 5980–5987. doi: 10.1021/la050277i 15952850

[14] Grunkemeier JM, Tsai WB, McFarland CD, Horbett TA. The effect of adsorbed fibrinogen, fibronectin, von Willebrand factor and vitronectin on the procoagulant state of adherent platelets. Biomaterials. 2000;21: 2243–2252. doi: 10.1016/s0142-9612(00)00150-2 11026630

[15] Johnell M, Larsson R, Siegbahn A. The influence of different heparin surface concentrations and antithrombin-binding capacity on inflammation and coagulation. Biomaterials. 2005;26: 1731–1739. doi: 10.1016/j.biomaterials.2004.05.029 15576147

[16] Johnell M, Elgue G, Larsson R, Larsson A, Thelin S, Siegbahn A. Coagulation, fibrinolysis, and cell activation in patients and shed mediastinal blood during coronary artery bypass grafting with a new heparin-coated surface. The Journal of Thoracic and Cardiovascular Surgery. 2002;124: 321–332. doi: 10.1067/mtc.2002.122551 12167793

[17] Kaseer H, Soto-Arenall M, Sanghavi D, Moss J, Ratzlaff R, Pham S, et al. Heparin vs bivalirudin anticoagulation for extracorporeal membrane oxygenation. J Card Surg. 2020;35: 779–786. doi: 10.1111/jocs.14458 32048330

[18] Macielak S, Burcham P, Whitson B, Abdel-Rasoul M, Rozycki A. Impact of anticoagulation strategy and agents on extracorporeal membrane oxygenation therapy. Perfusion. 2019;34: 671–678. doi: 10.1177/0267659119842809 31057056

[19] Linkins L-A, Julian JA, Rischke J, Hirsh J, Weitz JI. In vitro comparison of the effect of heparin, enoxaparin and fondaparinux on tests of coagulation. Thrombosis Research. 2002;107: 241–244. doi: 10.1016/s0049-3848(02)00340-7 12479885

[20] Parlar AI, Sayar U, Cevirme D, Yuruk MA, Mataraci I. Successful use of fondaparinux in a patient with heparin-induced thrombocytopenia while on extracorporeal membrane oxygenation after mitral valve redo surgery. Int J Artif Organs. 2014;37: 344–347. doi: 10.5301/ijao.5000302 24619895

[21] Andersson J, Sanchez J, Ekdahl KN, Elgue G, Nilsson B, Larsson R. Optimal heparin surface concentration and antithrombin binding capacity as evaluated with human non-anticoagulated bloodin vitro. J Biomed Mater Res. 2003;67A: 458–466. doi: 10.1002/jbm.a.10104 14566786

[22] Niimi Y, Ichinose F, Ishiguro Y, Terui K, Uezono S, Morita S, et al. The effects of heparin coating of oxygenator fibers on platelet adhesion and protein adsorption. Anesth Analg. 1999;89: 573–579. doi: 10.1097/00000539-199909000-00006 10475283

[23] Cornelius RM, Sanchez J, Olsson P, Brash JL. Interactions of antithrombin and proteins in the plasma contact activation system with immobilized functional heparin. J Biomed Mater Res. 2003;67A: 475–483. doi: 10.1002/jbm.a.10118 14566788

[24] Hirsh J, Raschke R. Heparin and low-molecular-weight heparin: the Seventh ACCP Conference on Antithrombotic and Thrombolytic Therapy. Chest. 2004;126: 188S–203S. doi: 10.1378/chest.126.3_suppl.188S 15383472

[25] Weitz JI, Fredenburgh JC, Eikelboom JW. A Test in Context: D-Dimer. Journal of the American College of Cardiology. 2017;70: 2411–2420. doi: 10.1016/j.jacc.2017.09.024 29096812

[26] Francis JL, Groce JB, Heparin Consensus Group. Challenges in variation and responsiveness of unfractionated heparin. Pharmacotherapy. 2004;24: 108S–119S. doi: 10.1592/phco.24.12.108s.36114 15334856

[27] Iwasaki Y, Uchiyama S, Kurita K, Morimoto N, Nakabayashi N. A nonthrombogenic gas-permeable membrane composed of a phospholipid polymer skin film adhered to a polyethylene porous membrane. Biomaterials. 2002;23: 3421–3427. doi: 10.1016/s0142-9612(02)00044-3 12099285

[28] Lewis AL, Furze JD, Small S, Robertson JD, Higgins BJ, Taylor S, et al. Long-term stability of a coronary stent coating post-implantation. J Biomed Mater Res. 2002;63: 699–705. doi: 10.1002/jbm.10387 12418013

[29] Messerer DAC, Vidoni L, Erber M, Stratmann AEP, Bauer JM, Braun CK, et al. Animal-Free Human Whole Blood Sepsis Model to Study Changes in Innate Immunity. Front Immunol. 2020;11: 571992. doi: 10.3389/fimmu.2020.571992 33178198 PMC7592114

[30] Hecke F, Hoehn T, Strauss E, Obladen M, Sonntag J. In-vitro activation of complement system by lactic acidosis in newborn and adults. Mediators Inflamm. 2001;10: 27–31. doi: 10.1080/09629350123788 11324901 PMC1781687

[31] Etulain J, Negrotto S, Carestia A, Pozner R, Romaniuk M, D’Atri L, et al. Acidosis downregulates platelet haemostatic functions and promotes neutrophil proinflammatory responses mediated by platelets. Thromb Haemost. 2012;107: 99–110. doi: 10.1160/TH11-06-0443 22159527

[32] Caspers M, Schäfer N, Fröhlich M, Bauerfeind U, Bouillon B, Mutschler M, et al. How do external factors contribute to the hypocoagulative state in trauma-induced coagulopathy?—In vitro analysis of the lethal triad in trauma. Scand J Trauma Resusc Emerg Med. 2018;26: 66. doi: 10.1186/s13049-018-0536-8 30111342 PMC6094881

[33] Coppell JA, Thalheimer U, Zambruni A, Triantos CK, Riddell AF, Burroughs AK, et al. The effects of unfractionated heparin, low molecular weight heparin and danaparoid on the thromboelastogram (TEG): an in-vitro comparison of standard and heparinase-modified TEGs with conventional coagulation assays. Blood Coagul Fibrinolysis. 2006;17: 97–104. doi: 10.1097/01.mbc.0000203859.62739.25 16479191

[34] Thomas O, Larsson A, Tynngård N, Schött U. Thromboelastometry versus free-oscillation rheometry and enoxaparin versus tinzaparin: an in-vitro study comparing two viscoelastic haemostatic tests’ dose-responses to two low molecular weight heparins at the time of withdrawing epidural catheters from ten patients after major surgery. BMC Anesthesiol. 2015;15: 170. doi: 10.1186/s12871-015-0145-2 26603039 PMC4659161

[35] Godier A, Durand M, Emmerich J, Dizier B, Lecompte T, Samama C-M. Efficacy of prothrombin complex concentrate to reverse the anticoagulant effect of the pentasaccharide fondaparinux in a rabbit model. Thromb Haemost. 2011;105: 161–168. doi: 10.1160/TH10-07-0434 20941458

[36] Eller T, Busse J, Dittrich M, Flieder T, Alban S, Knabbe C, et al. Dabigatran, rivaroxaban, apixaban, argatroban and fondaparinux and their effects on coagulation POC and platelet function tests. Clin Chem Lab Med. 2014;52: 835–844. doi: 10.1515/cclm-2013-0936 24406289

[37] Salta S, Papageorgiou L, Larsen AK, Van Dreden P, Soulier C, Cokkinos DV, et al. Comparison of antithrombin-dependent and direct inhibitors of factor Xa or thrombin on the kinetics and qualitative characteristics of blood clots. Res Pract Thromb Haemost. 2018;2: 696–707. doi: 10.1002/rth2.12120 30349889 PMC6178701

[38] Arantes FBB, Menezes FR, Franci A, Barbosa CJDG, Dalçoquio TF, Nakashima CAK, et al. Influence of Direct Thrombin Inhibitor and Low Molecular Weight Heparin on Platelet Function in Patients with Coronary Artery Disease: A Prospective Interventional Trial. Adv Ther. 2020;37: 420–430. doi: 10.1007/s12325-019-01153-8 31758517 PMC6979460

[39] Balle CM, Jeppesen AN, Christensen S, Hvas A-M. Platelet Function During Extracorporeal Membrane Oxygenation in Adult Patients. Front Cardiovasc Med. 2019;6: 114. doi: 10.3389/fcvm.2019.00114 31440518 PMC6694790

[40] Wand S, Huber-Petersen JF, Schaeper J, Binder C, Moerer O. Platelet Function Disturbance During Veno-Venous ECMO in ARDS Patients Assessed by Multiple Electrode Aggregometry-A Prospective, Observational Cohort Study. J Clin Med. 2019;8. doi: 10.3390/jcm8071056 31330966 PMC6678447

[41] Aggarwal A, Sobel BE, Schneider DJ. Decreased platelet reactivity in blood anticoagulated with bivalirudin or enoxaparin compared with unfractionated heparin: implications for coronary intervention. J Thromb Thrombolysis. 2002;13: 161–165. doi: 10.1023/a:1020478923794 12355033

[42] Aggarwal A, Whitaker DA, Rimmer JM, Solomon RJ, Gennari FJ, Sobel BE, et al. Attenuation of platelet reactivity by enoxaparin compared with unfractionated heparin in patients undergoing haemodialysis. Nephrology Dialysis Transplantation. 2004;19: 1559–1563. doi: 10.1093/ndt/gfh209 15034156

[43] Mann KG, Whelihan MF, Butenas S, Orfeo T. Citrate anticoagulation and the dynamics of thrombin generation. J Thromb Haemost. 2007;5: 2055–2061. doi: 10.1111/j.1538-7836.2007.02710.x 17883701

[44] Schneider David J., Tracy Paula B., Mann Kenneth G., Sobel Burton E. Differential Effects of Anticoagulants on the Activation of Platelets Ex Vivo. Circulation. 1997;96: 2877–2883. doi: 10.1161/01.cir.96.9.2877 9386152

[45] Bernhard S, Hug S, Stratmann AEP, Erber M, Vidoni L, Knapp CL, et al. Interleukin 8 Elicits Rapid Physiological Changes in Neutrophils That Are Altered by Inflammatory Conditions. J Innate Immun. 2021; 1–17. doi: 10.1159/000514885 33857948 PMC8460987

[46] Hakimi J, Aboutorabian S, To F, Ausar SF, Rahman N, Brookes RH. Screening Vaccine Formulations in Fresh Human Whole Blood. In: Fox CB, editor. Vaccine Adjuvants: Methods and Protocols. New York, NY: Springer New York; 2017. pp. 295–304. 10.1007/978-1-4939-6445-1_22 27718203

[47] Halbgebauer R, Kellermann S, Schäfer F, Weckbach S, Weiss M, Barth E, et al. Functional immune monitoring in severely injured patients—A pilot study. Scand J Immunol. 2020;91. doi: 10.1111/sji.12837 31622512

[48] Sangli SS, Noronha SF, Mourad B, Jean R, Bohman JK, Seelhammer TG. A Systematic Review of Preexisting Sepsis and Extracorporeal Membrane Oxygenation. ASAIO Journal. 2020;66: 1–7. doi: 10.1097/MAT.0000000000000934 31860607

[49] Gopalakrishnan R, Vashisht R. Sepsis and ECMO. Indian J Thorac Cardiovasc Surg. 2021;37: 267–274. doi: 10.1007/s12055-020-00944-x 32421057 PMC7223121

